# 

**Published:** 1863-08

**Authors:** 


### BACK NUMBERS.

The January number of the present (17th) volume is entirely exhausted, and we desire to obtain a few numbers. Those having copies of that number, that they are not anxious to preserve, will please mail them to us and we will send them three or four of any other numbers they may desire, that we now have or may have.

To those who have recently subscribed for the 17th volume, and have not received the first, this will afford sufficient explanation; and we desire all such who wish to keep complete files of the *Register* to notify us, and we will endeavor to supply them.

J. TAFT.

